# GutAlive^®^ enables DNA-based microbiome analysis without disrupting the original composition and diversity

**DOI:** 10.3389/fmicb.2023.1118291

**Published:** 2023-04-06

**Authors:** Ignacio Montero, Desirée Barrientos, Claudio Hidalgo-Cantabrana, Noelia Martínez-Álvarez

**Affiliations:** MicroViable Therapeutics SL, Gijón, Spain

**Keywords:** fecal microbiome sampling, microbiome analysis, protocol normalization, shotgun metagenomics, functional profiling

## Abstract

**Introduction:**

A precise fecal microbiome analysis requires normalized methods for microbiome sampling, transport and manipulation in order to obtain a representative snapshot of the microbial community. GutAlive^®^ is the unique stool collection kit that generates an anaerobic atmosphere enabling oxygen sensitive bacteria to survive, maintaining the original microbiome composition and diversity.

**Methods:**

Five stool samples from different donors were collected using two different sampling devices, GutAlive^®^ and Zymo DNA/RNA Shield^®^, and processed at four different time points. Shotgun metagenomics was used to evaluate the influence of the device and the processing timing on the microbial populations to unravel the potential fluctuations on the composition and diversity of the fecal microbiome and the metabolic pathways profiling. Additionally, RT-qPCR was used to quantify bacterial cell viability for downstream applications of microbiota samples beyond metagenomics.

**Results:**

Our results show that GutAlive^®^ enables bacterial cell viability overtime preserving DNA integrity, obtaining high-quantity and high-quality DNA to perform microbiome analysis using shotgun metagenomics. Based on the taxonomic profiling, metabolic pathways analysis, phylogeny and metagenome-assembled genomes, GutAlive^®^ displayed greater performance without significant variability over time, showcasing the stabilization of the microbiome preserving the original composition and diversity. Indeed, this DNA stabilization is enabled with the preservation of bacterial viability on an anaerobic environment inside of the sampling device, without the addition of any reagents that interact directly with sample.

**Conclusion:**

All the above makes GutAlive^®^ an user-friendly kit for self-collection of biological samples, suitable for microbiome analysis, diagnostics, fecal microbiota transplant and bacterial isolation, maintaining the stability and bacterial viability over time, preserving the original composition and diversity of the microbiome.

## 1. Introduction

The human body harbors a diverse community of microorganisms that live in a symbiotic relationship with the host. In particular, the gut microbiota performs essential physiological functions, such as preventing infection by various pathogens (Kamada et al., 2013; Andoh, 2016; George et al., 2022); participating in the maturation and regulation of the immune response (Zheng et al., 2020); production of essential compounds (vitamins, amino acids, and neurotransmitters), nutrient absorption, and metabolism (Rowland et al., 2018; Oliphant and Allen-Vercoe, 2019); maintenance of mental health (Tremlett et al., 2017; Castillo-Álvarez and Marzo-Sola, 2022); and promotion of anti-cancer functions (Roy and Trinchieri, 2017; Helmink et al., 2019), among others.

Comprehensive knowledge of the taxa, relative abundance, metabolic pathways, and genetic characteristics are key elements for understanding the microbial dynamics and crosstalk interactions with the host. Over the last few years, extensive efforts have focused on the characterization of the human microbiome, based on sequencing technologies coupled with bioinformatics pipelines to decode the correlation between specific bacterial taxa and health status (Qian et al., 2020; Gao et al., 2021).

In contrast to other environments, such as the oral, skin, or vaginal microbiome, obtaining samples of the gut microbiota is a major challenge in the field. Even though different sections of the gastrointestinal tract harbor distinct microbiota, fecal samples have become the gold-standard unit of sampling to avoid invasive procedures (Kumar et al., 2014; Thomas et al., 2015; Tang et al., 2020). Despite attempts at standardization by institutions such as the International Human Microbiome Standards (2015) and the Spanish Association of Gastroenterology (García García de Paredes et al., 2019), procedures to collect and store fecal samples remain largely unnormalized in published studies and could lead to important differences between them.

Accurate and precise fecal microbiome analysis requires reliable and normalized methods of microbiome sampling, transport, and manipulation to obtain a representative snapshot of the microbial community contained in each sample at the time of collection (Thomas et al., 2015; Tang et al., 2020). To date, there are several commercially available kits from various vendors that mainly focus on nucleic acid stabilization using specific buffer solutions. However, these kits have a limited volume size, which introduces bias to the self-collection sampling process performed by the donor and limits the amount of sample collected. Moreover, these commercial kits neither consider microbial viability for downstream isolation of bacteria of interest nor do they account for reliable sample conservation for fecal material transplant procedures (Thomas et al., 2015).

Previously, we have shown that our sampling device, GutAlive^®^, is a unique microbiome collection kit that generates an anaerobic atmosphere, enabling oxygen-sensitive bacteria to survive (Martínez et al., 2019). In this study, we have tested whether GutAlive^®^ is suitable for microbiome analysis, maintaining the original composition and diversity of the microbiota over time. We demonstrate that, in addition to maintaining microbial viability, GutAlive^®^ stabilizes the fecal microbiome community over time—at room temperature—enabling microbiome analysis through shotgun metagenomics and other DNA-based methods.

Overall, we have demonstrated that GutAlive^®^ enables the standardization of sample collection and transport, without refrigeration, minimizing variations among procedures and contributing to protocol normalization. Moreover, maintaining the original composition and diversity of the microbiome makes this commercial device suitable for various applications such as microbiome analysis, diagnostics, and other downstream applications such as isolation of anaerobic bacteria with therapeutic potential, microbiota-based therapeutics, and fecal microbiota transplantation.

## 2. Materials and methods

### 2.1. Ethical statements, donor recruitment, and sample collection

This project was conducted using fecal samples from four healthy donors (one man and three women; age range between 31 and 47 years). Subject recruitment was carried out at Microviable Therapeutics SL (Asturias, Spain). Exclusion criteria were having undergone any medical treatment with antibiotics or glucocorticoids in the previous 3 months. Volunteers were informed of the objectives of the study, and all samples were collected and analyzed with the fully informed, written consent of all participants involved in the study and with the approval of the Ethics Committee. Samples were anonymized using an alphanumeric code.

Ethics approval (reference CEImPA: 2020-024) for this study was obtained from the Regional Ethics Committee for Clinical Research (Comité de Ética de Investigación del Principado de Asturias), in compliance with the Declaration of Helsinki. Personal data and fecal samples were collected according to the ethical guidelines of the Declaration of Helsinki. Informed consent was obtained from all donors prior to inclusion in the study.

**Figure 1:**
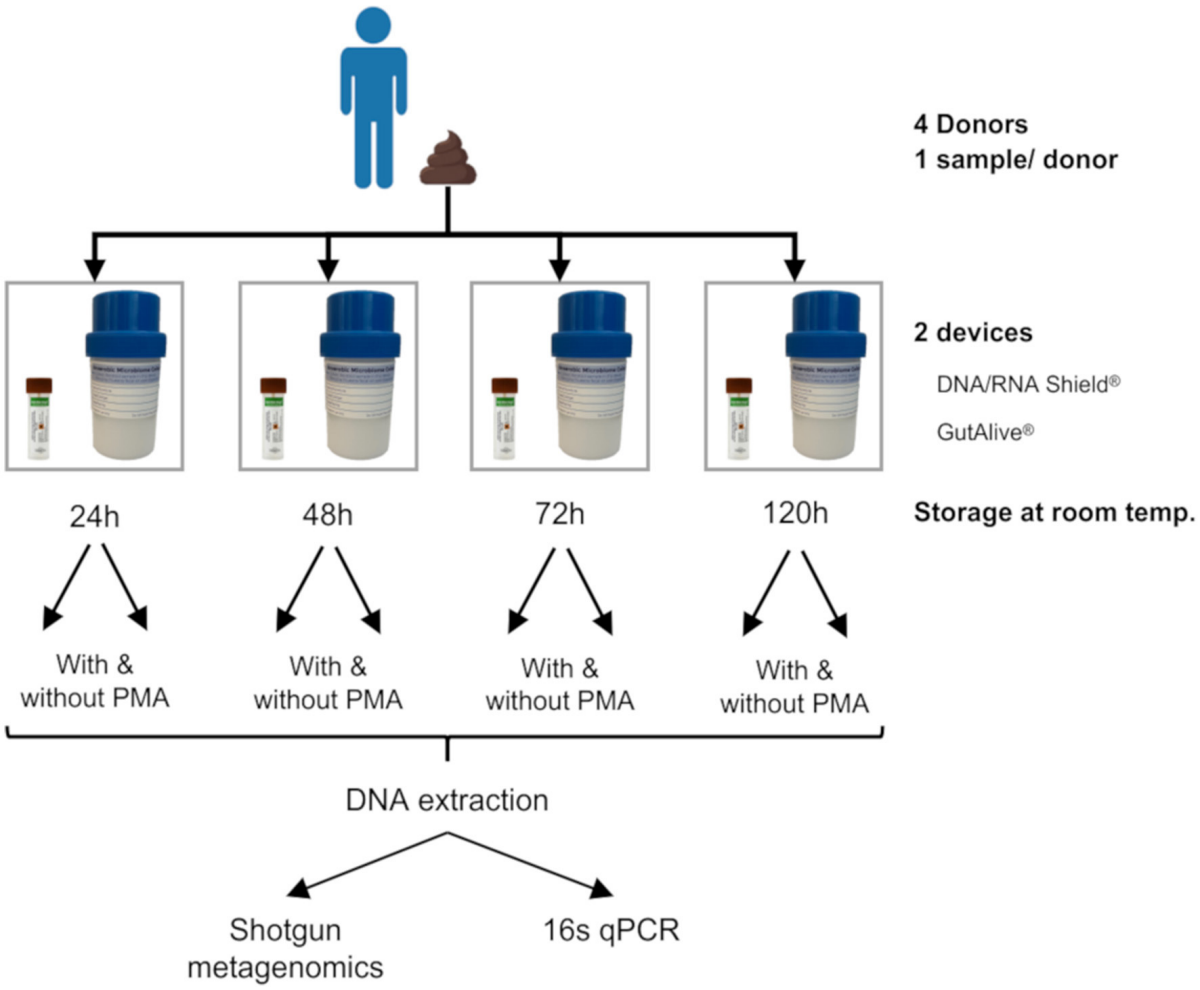
Experimental design. One stool sample was self-collected by each donor (with a total of four donors). Each fecal sample was divided into eight subsamples by the donor at the time of deposition, four of which were collected with DNA/RNA Shield^®^ and the other four with GutAlive^®^. Samples were processed at 24, 48, 72, and 120 h at room temperature, treated with or without PMA. The DNA was extracted from each sample to perform shotgun metagenomics and 16s qPCR.

The stool samples were collected from each donor. One fecal sample per donor was divided into eight different subsamples for self-collection using GutAlive^®^ (Microviable Therapeutics, Spain) and DNA/RNA Shield^®^ (Zymo Research, United States) kits. All the samples were shipped to Microviable's laboratory facilities at room temperature and received within 24 h of collection. The samples were kept at room temperature (15°C−20°C) and processed at 24, 48, 72, and 120 h after collection (Figure 1).

### 2.2. DNA extraction

For each GutAlive^®^ sample and time point, 600 mg of feces were homogenized and divided into two aliquots of 300 mg for DNA extraction, with and without the propidium monoazide (PMA) treatment. PMA allows differentiation between intracellular DNA (from viable bacteria) and extracellular free DNA (dead bacterial cells) in the subsequent PCR amplifications. Briefly, for the PMA-treated aliquot, 300 mg of feces were resuspended in 800 μl of PBS and 2 μl of PMA were added, followed by 10' incubation in darkness, 30' incubation under blue light, and 5' centrifugation at 15,000 rpm. Then, DNA extraction was performed using QIAamp^®^ PowerFecal^®^ Pro DNA Kit (Qiagen).

Samples collected in DNA/RNA Shield^®^ (1 gram of feces in 900 μl of buffer) were processed following the manufacturer's instructions for DNA extraction with the QIAamp^®^ PowerFecal^®^ Pro DNA Kit using 300 μl of fecal suspension. PMA treatment was not applied to these samples to test bacterial viability, as these commercial kits are only designed to preserve DNA integrity.

All extracted DNA samples were quantified with Nanodrop.

### 2.3. Real-time qPCR

The extracted DNA was amplified using 16s rRNA real-time PCR (qPCR) to validate PCR efficiency, detect potential carryover inhibitors, and compare bacterial viability. The primers used were qPCR1369-F (5′CGGTGAATACGTTCCCGG3′) and qPCR1492-R (5′TACGGCTACCTTGTTACGACTT3′) (Suzuki et al., 2000). The correlation between DNA quantity and total cells or viable cells (cfu/gr) was made using *Escherichia coli* DNA for the standard curve. Experiments were performed in triplicate, with 10-fold serial dilutions ranging from 10^3^ colony-forming units (cfu) to 10^7^ cfu of *E. coli* DNA to generate the standard curves. All real-time PCR runs were performed on the HT7900 Real-Time PCR equipment (Applied Biosystems), with a standard mode default protocol. The amplification reaction was made in a final volume of 20 μl containing 1 × Power Syber^®^ Green PCR Master Mix (Thermo Scientific), 300 nM of each oligonucleotide, and 0.5 ng of total DNA, PMA-treated or not, from each sample and collection time point, in triplicate. Equal amounts of DNA from each sample and time point were used, and the correlation with ng of DNA and grams of fecal samples was calculated.

The Ct values were obtained using Sequence Detection System (SDS) v2.3 software (Applied Biosystems), and the cfu was calculated from the y-intercept and slope of the standard curve, as follows: Sample cell count = [(Ct – y-intercept) / slope].

Since PMA allows differentiation between intracellular DNA (viable bacteria) and extracellular free DNA (from dead bacterial cells) at the time of DNA extraction, it allows quantification of the loss of bacterial viability in the samples collected with GutAlive^®^.

### 2.4. Microbiome analysis

The DNA samples were sequenced using shotgun metagenomics on an Illumina HiSeq 2,500 with paired-end 150 bp reads (average of 20 million reads per sample) at Eurofins Scientific (Ebersberg, Germany). Fastq files containing the resulting reads were quality-filtered and cleaned of contaminating reads from the host using KneadData 0.10.0 with default parameters (McIver et al., 2018). Subsequently, the number of sequences to be used was rarefied and adjusted to the minimum number of sequences to avoid bias due to the different quantity of information (number of sequences) per sample.

Taxonomic assignment was performed using MetaPhlAn 3.0.7 (09 December 2020) with default parameters and the CHOCOPhlan v201901 database (McIver et al., 2018). Metabolic pathway profiling was analyzed using HUMAnN 3.0.0.alpha.4 with default parameters and the Uniref90 v201901 database (McIver et al., 2018). Strain-level characterization of identified taxa was performed using StrainPhlAn 3.0 (1 September 2020) and PhyloPhlAn 3.0.67 (24 August 2022) (Caspi et al., 2018; Beghini et al., 2021).

Metagenome-assembled genomes (MAGs) were assembled using MEGAHIT v.1.2.9 (Li et al., 2016) with default settings, except for the maximum k-mer size, set at 141, generating a series of k-mers with different lengths that are shorter than entire reads. MEGAHIT results were grouped and assigned to individual genomes in the binning step performed using the Autometa v.2.1.0 bash workflow (Miller et al., 2019). Quality control of the obtained bins was assessed using CheckM v.1.2.1 lineage-specific workflow, the recommended workflow for assessing the completeness and contamination of genome bins (Parks et al., 2015). MAGs showing completeness higher than 50% and contamination lower than 5% were selected according to Asnicar et al. (2021). Taxonomic identification of MAGs was performed with GTDB-Tk v.2.1.1 (Chaumeil et al., 2020), and functional profiling was carried out using Prodigal v.2.6.3 (Hyatt et al., 2010), Kofam_scan v.1.3.0 (Aramaki et al., 2020), and KEGGDecoder v.1.3 (Graham et al., 2018) with default settings.

Statistical analysis of alpha and beta divergences was performed using the Adonis, Kruskal–Wallis, and Analysis of Composition of Microbiomes tests implemented in packages within R-Studio 2022.02.1+461 for the Ubuntu 22.04.3 LTS environment. Graphical representation of PCoA, heatmaps, and bar plots was performed using the phyloseq, microViz, and ggplot2 packages, respectively, within R-Studio 2022.02.1+461 for the Ubuntu 22.04.3 LTS environment.

### 2.5. Data availability

The raw shotgun metagenomic sequences reported in this article have been deposited in the NCBI SRA repository and are accessible under BioProject accession number PRJNA909041.

## 3. Results

### 3.1. DNA stability and bacterial viability

A total of four stool samples were collected from healthy donors and stored at room temperature for 24, 48, 72, and 120 h in both sampling devices to analyze DNA stability over time (Figure 1). High-yield and high-quality DNA was obtained from both the DNA/RNA Shield^®^ and GutAlive^®^ collection devices, independent of each donor and sampling point.

The average difference between the measurement of total bacteria detected in GutAlive^®^ samples and the viable bacteria quantified by qPCR using 16S rRNA primers, was 0.10 ± 0.34 log units (mean ± sd), displaying no significant differences. Moreover, no significant differences were observed over the 5 days of the study. A minor reduction of 0.4 log units in bacterial viability was detected after 5 days of storage in GutAlive^®^ at room temperature.

### 3.2. Microbiome analysis

Microbiome analysis based on shotgun metagenomics was performed at different time points to test the performance of both sample collection devices and the impact on microbiome composition, diversity, and stability over time.

**Figure 2:**
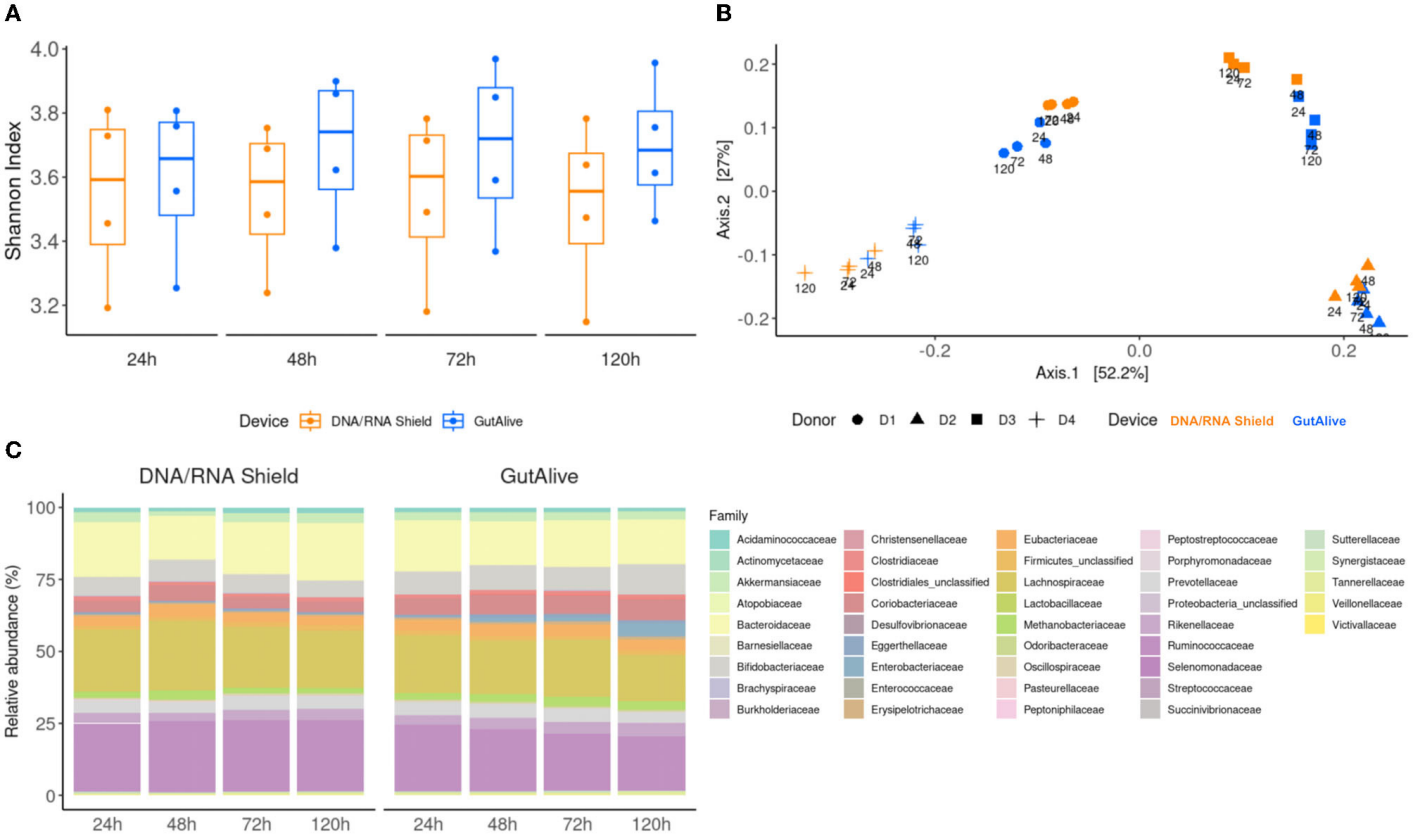
Taxonomic profiling and alpha and beta divergences. **(A)** Shannon index. **(B)** PCoA of Bray–Curtis distance. **(C)** Identified bacterial taxa (family level) and relative abundance.

The alpha diversity, quantified with the Shannon index (Figure 2A), did not show significant differences between the sampling devices or the different time points (*p* > 0.05), indicating that bacterial diversity was stable over the 5 days of the study for both devices. Similar results were obtained with other alpha diversity indices, such as Chao, Simpson, Inverted Simpson, and Fisher (data not shown). The beta diversity analysis based on the Bray–Curtis dissimilarity index, coupled with principal coordinate analysis, displayed differential clustering of the samples based on the donor (Adonis *p* < 0.001) and the collection device (Adonis *p* < 0.004). No significant influence was detected based on the processing time point (Figure 2B).

To further visualize potential microbiome fluctuations, the identified bacterial taxa, and their relative abundance were represented at several taxonomic levels for each device and time point, as an average of the four donors (Figure 2C). The ANCOM statistical analysis displayed no significant differences in the relative abundance of the identified taxa over the 5 days of the study, reflecting microbiome stabilization.

**Figure 3:**
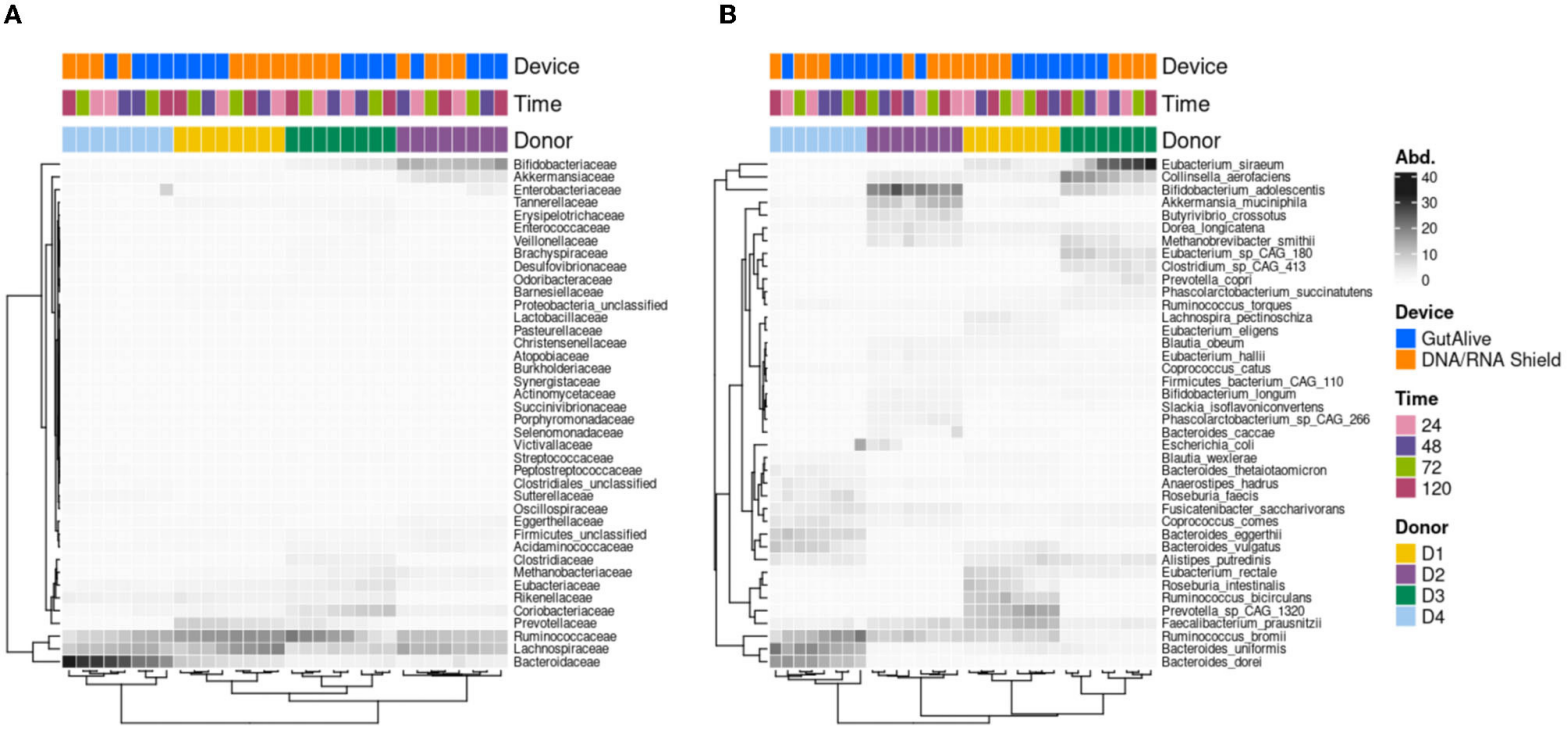
Heatmap of the abundance of the top 40 taxa. **(A)** Family level. **(B)** Species level.

Similarly, when the top 40 most abundant bacterial families and species were represented on a heat map, no influence of the sampling time point was observed, with significant clustering based on the donor (Figure 3).

**Figure 4:**
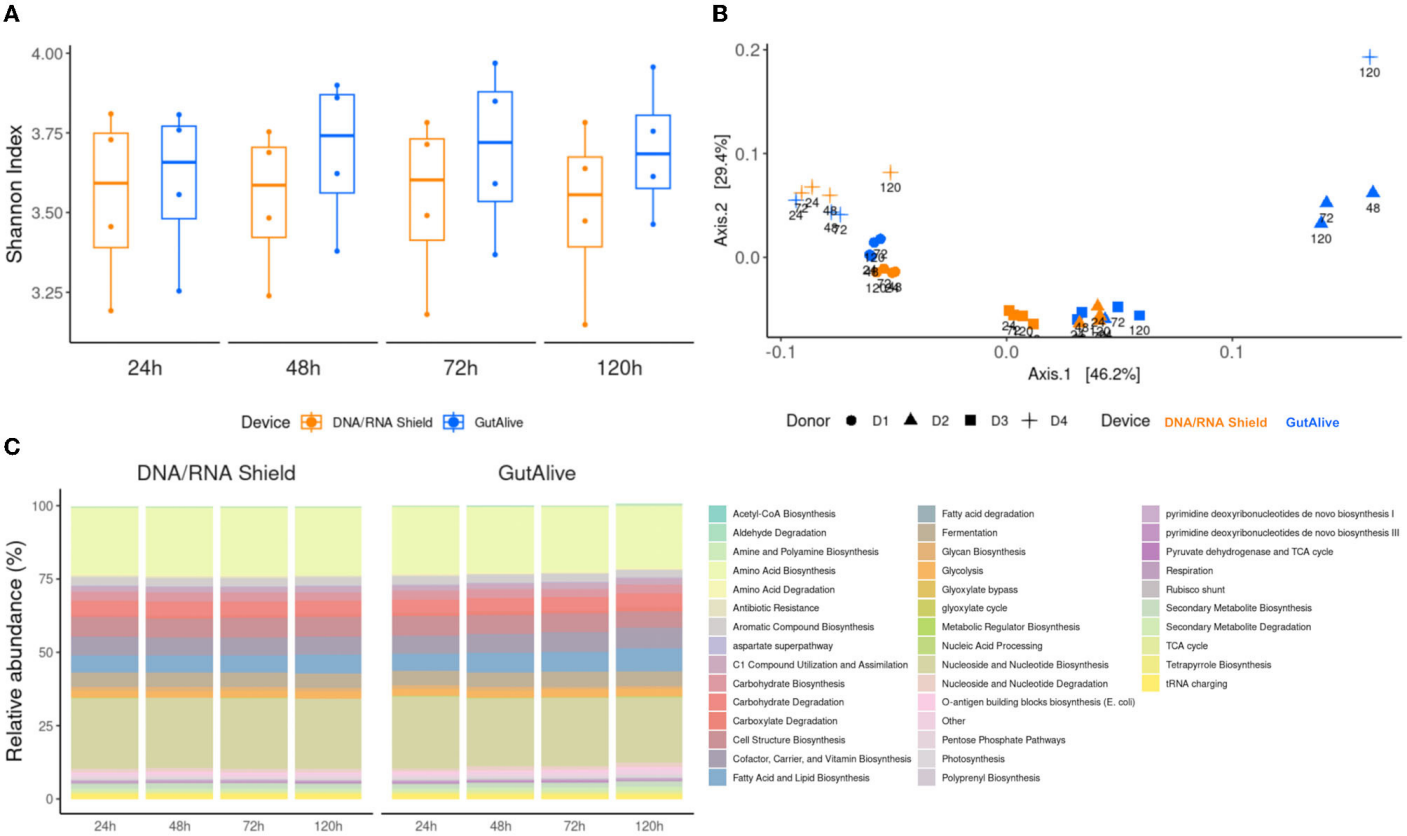
Functional profiling and alpha and beta divergences. **(A)** Shannon index. **(B)** PCoA of Bray–Curtis distance. **(C)** Functional categories of cross-sectional study functionalities of the identified metabolic pathways and relative abundance.

Consistent with the taxonomy results, the functional analysis did not reveal any significant differences for the metabolic pathways identified based on the alpha diversity, for the sampling devices, nor the different time points, indicating that bacterial diversity was stable over the 5 days of the study for both devices (Figure 4A). Principal coordinate analysis of Bray–Curtis distances still showed differential clustering of the samples based on the donor microbiome profiling (Adonis *p* < 0.001), despite it not being as specific as in the taxonomy (Figure 4B). Moreover, the identified metabolic pathways grouped into functional categories did not show significant differences using ANCOM, as shown in Figure 4C.

**Figure 5:**
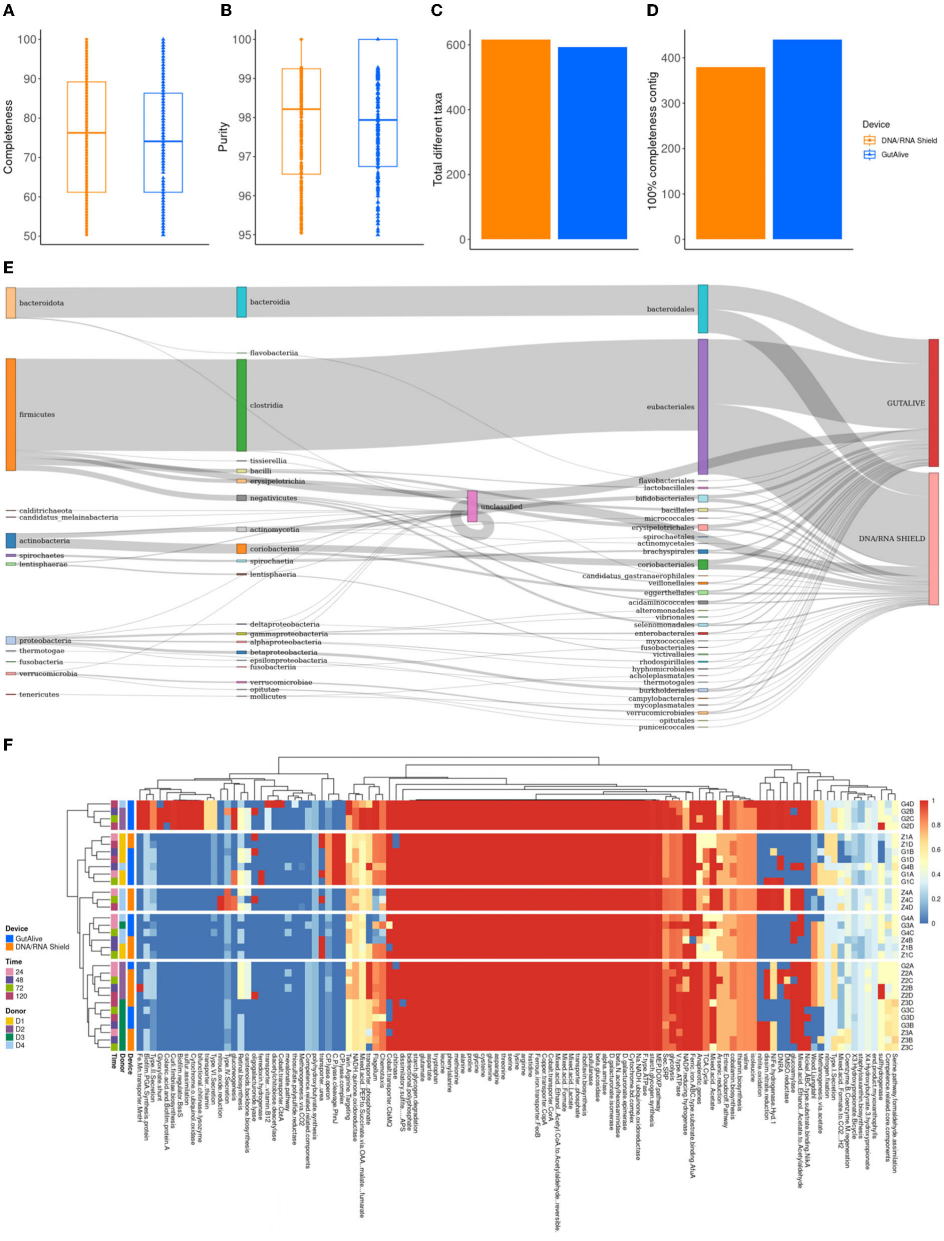
Metagenome-assembled genome analysis. **(A)** Completeness. **(B)** Purity. **(C)** Total number of different taxa. **(D)** Number of contigs with 100% completeness. **(E)** Sankey plot from phylum to family-level. **(F)** Heatmap representing the functional categories of the identified metabolic pathways.

To derive deeper information from shotgun metagenomics sequencing and to compare potential biases in the results based on the collection device or sampling time point, metagenome-assembled genomes (MAGs) were constructed for all the samples. The completeness level of the MAGs was equivalent for both devices (74.0 % ± 15.1 % and 75.5 % ± 15.1 %, GutAlive^®^ and DNA/RNA Shield^®^, respectively) (Figure 5A), as was the purity (98.0 % ± 1.6 % and 97.9 % ± 1.7 %) (Figure 5B) and the number of unique taxa recognized (593 and 616, respectively) (Figure 5C). Notably, GutAlive^®^ provided 440 complete MAGs while DNA/RNA Shield^®^ returned 381 (Figure 5D).

The taxonomic classification of the MAGs did not show significant differences between devices (Figure 5E) and sample time points although there were differences between donors (Adonis *p* < 0.001). Functional profiling at the level of MAGs was consistent with the above, showing no differences between the variables studied (Figure 5F).

## 4. Discussion

The human microbiome is one of the fastest-growing areas of biomedical research, from diagnostics to therapeutics. However, there is a lack of consistency in protocol normalization, which definitely affects the comparability of results between different studies. The standardization of sample collection, DNA extraction, and bioinformatics analysis of sequencing data is lagging behind (Cardona et al., 2012; Santiago et al., 2014; International Human Microbiome Standards, 2015). In this study, we propose the use of GutAlive^®^ as a standard method to collect stool samples and maintain their quality over time for further downstream analysis. It has been demonstrated that GutAlive^®^ maintains the viability of strict anaerobes typically present in fecal samples due to the anaerobic atmosphere generated within the collection device (Martínez et al., 2019). In this study, we demonstrate the usability of GutAlive^®^ for DNA-based microbiome analysis, allowing shotgun metagenomics to be performed at different time points without disrupting the original microbiome composition and diversity and maintaining bacterial viability and DNA stability over time.

Commercially available stool collection devices can only accommodate a few grams of sample, introducing several limitations on sample quantity and sample availability, especially when various applications or replicates are required. In addition, the extensive donor recruitment efforts required in any project can be rendered useless if not enough sample is collected. In addition to microbiome analysis and diagnostics, fecal bacterial isolation or fecal microbiota transplantation will require higher amounts of samples (Cammarota et al., 2017) and bacterial cells in viable conditions. GutAlive^®^ can accommodate up to 120 g, which is less restrictive on the amount collected (Wang et al., 2018) and an advantage, especially when several applications are going to be performed on the same sample. Moreover, the anaerobic atmosphere generated inside ensures the survival of oxygen-sensitive bacteria, including strict anaerobic bacteria (Martínez et al., 2019), which allows for various downstream applications, including microbiome analysis, bacterial isolation, cryopreservation, and/or fecal microbiota transplantation. In addition to this high-capacity container, there is no bias introduced during donor self-collection of the sample, as the entire deposition can be collected.

Regarding sample processing times, it is important to highlight that in most cases, there is a delay between sample collection and laboratory processing, with some protocols introducing a freezing step [−20°C or −80°C] (Cardona et al., 2012; Santiago et al., 2014; International Human Microbiome Standards, 2015; Alarcón Cavero et al., 2016; Cammarota et al., 2017; Wang et al., 2018; Bellali et al., 2019); however, this can extremely affect bacterial viability. GutAlive^®^ showed great performance in stabilizing samples at room temperature without the need for refrigeration, reducing logistics costs and microbiome alteration, and was recommended by the Spanish Association of Gastroenterology (García García de Paredes et al., 2019).

Microbiome analysis and diagnostics require high-quality DNA to obtain a representative snapshot of the microbial community. There are many user-friendly and small kits designed for this purpose, and one example is DNA/RNA Shield^®^, which uses buffer solutions in contact with the sample to stabilize nucleic acids. GutAlive^®^ is able to enhance DNA stability by preserving bacterial viability and generating anaerobic conditions inside the device without adding any reagents to the sample that may alter it for other downstream applications and not just for DNA extraction. We used PMA coupled with qPCR to obtain accurate bacterial quantification and the absence of differences between viable bacteria and total bacteria shows that GutAlive^®^ preserves bacterial viability and therefore, bacterial DNA integrity, making it a suitable device for DNA-based microbiome analysis. The shotgun metagenomic analysis enables microbiome profiling and functional analysis to elucidate the bacterial taxa that play a key role in human health and the dysbiosis associated with certain diseases. A truly representative sample is required for accurate diagnosis and any potential bias in microbial populations due to sample processing time and DNA degradation, and other variables and fluctuations should be avoided. In this regard, GutAlive^®^ showed great performance for microbiome analysis over the 5 days of the study, maintaining consistency in bacterial taxa and their relative abundance. GutAlive^®^ was able to maintain sample stability over time, capturing a snapshot of the original microbiome composition and diversity, representing a fingerprint of each donor, with no alterations (alpha and beta divergences) in the microbial populations.

M microbiome functional analysis based on pathway identification provided consistent results across variables, with similar conclusions to the taxonomic profiling in terms of sample stability over time. The significant donor clustering observed with taxonomic profiling was not as distinct from metabolic pathways, mainly because of the broad core metabolic functions shared among different bacterial taxa (Turnbaugh et al., 2009).

Notably, the MAG analysis showed that GutAlive^®^-collected samples can yield genomes with a level of purity and completeness comparable to that of DNA/RNA Shield^®^. The taxonomic and functional profiling based on MAGs confirmed the results of the aforementioned metagenomic analysis, with the composition and diversity of the microbiome remaining consistent over the 5 days of the study.

All these data allow us to conclude that GutAlive^®^ is a high-capacity, user-friendly kit for the self-collection of biological samples under anaerobic conditions, allowing microbiome stabilization based on bacterial cell viability. These characteristics make GutAlive^®^ suitable for DNA-based microbiome analysis and diagnostics, opening new avenues for protocol normalization and method standardization, but also for other potential applications that require live cells, such as fecal microbiota transplantation or anaerobic bacterial isolation for the development of live biotherapeutic products.

## Data availability statement

The datasets presented in this study can be found in online repositories. The names of the repository/repositories and accession number(s) can be found below: BioProject ID: PRJNA909041 (to be released if accepted).

## Ethics statement

The studies involving human participants were reviewed and approved by Comité de Ética de la Investigación del Principado de Asturias (reference: CEImPA 2020-024). The patients/participants provided their written informed consent to participate in this study.

## Author contributions

IM performed the bioinformatics and statistical analyses. DB performed the laboratory experiments. NM-Á and CH-C supervised the project. All authors approved the final version of the manuscript.
